# Regional heritability mapping identifies several novel loci (*STAT4, ULK4*, and *KCNH5*) for primary biliary cholangitis in the Japanese population

**DOI:** 10.1038/s41431-021-00854-5

**Published:** 2021-04-09

**Authors:** Olivier Gervais, Kazuko Ueno, Yosuke Kawai, Yuki Hitomi, Yoshihiro Aiba, Mayumi Ueta, Minoru Nakamura, Katsushi Tokunaga, Masao Nagasaki

**Affiliations:** 1 Center for the Promotion of Interdisciplinary Education and Research, Kyoto University, Sakyo-ku, Kyoto Japan; 2 Center for Genomic Medicine, Kyoto University, Sakyo-ku, Kyoto Japan; 3 Tohoku Medical Megabank Organization, Tohoku University, Sendai, Miyagi Japan; 4 Department of International Studies, College of International Relations, Nihon University, Mishima, Shizuoka Japan; 5 Genome Medical Science Project, National Center for Global Health and Medicine, Shinjuku, Tokyo Japan; 6 Department of Human Genetics, Graduate School of Medicine, The University of Tokyo, Bunkyo, Tokyo Japan; 7 Department of Microbiology, Hoshi University School of Pharmacy and Pharmaceutical Sciences, Shinagawa, Tokyo Japan; 8 Clinical Research Center, National Hospital Organization (NHO) Nagasaki Medical Center, Omura, Nagasaki Japan; 9 Department of Frontier Medical Science and Technology for Ophthalmology, Kyoto Prefectural University of Medicine, Kamigyo-ku, Kyoto Japan; 10 Department of Hepatology, Nagasaki University Graduate School of Biomedical Sciences, Omura, Nagasaki Japan

**Keywords:** Quantitative trait, Genome informatics

## Abstract

While the advent of GWAS more than a decade ago has ushered in remarkable advances in our understanding of complex traits, the limitations of single-SNP analysis have also led to the development of several other approaches. Simulation studies have shown that the regional heritability mapping (RHM) method, which makes use of multiple adjacent SNPs jointly to estimate the genetic effect of a given region of the genome, generally has higher detection power than single-SNP GWAS. However, thus far its use has been mostly limited to agricultural settings, and its potential for the discovery of new genes in human diseases is yet to be fully exploited. In this study, by applying the RHM method to primary biliary cholangitis (PBC) in the Japanese population, we identified three novel loci (*STAT4, ULK4*, and *KCNH5*) at the genome-wide significance level, two of which (*ULK4* and *KCNH5*) have not been found associated with PBC in any population previously. Notably, these genes could not be detected by using conventional single-SNP GWAS, highlighting the potential of the RHM method for the detection of new susceptibility loci in human diseases. These findings thereby provide strong empirical evidence that RHM is an effective and practical complementary approach to GWAS in this context. Also, liver tissue mRNA microarray analysis revealed higher gene expression levels in *ULK4* in PBC patients (*P* < 0.01). Lastly, we estimated the common SNP heritability of PBC in the Japanese population (0.210 ± 0.026).

## Introduction

Since the publication of the first GWAS study at the beginning of the twenty-first century, thousands of GWAS analyses have been performed and published; however, with the improvement of genotyping technologies, increased sophistication in study design, and the development of large-scale DNA biobanks and cohorts containing a wide range of clinical data/phenotypes on hundreds of thousands of individuals, the genome-wide association studies conducted nowadays are often much more elaborate than they were only a decade ago.

In spite of the many successes of GWAS, this development has created a need for innovative analytical methods and statistical models to better make sense of this newly available data, for instance by accounting for population structure and relatedness, reducing error rates in unbalanced case/control traits, improving the detection power of rare variants, or analyzing complex immune-mediated diseases [1]. These aspects are especially important given that the heterogeneous nature of the genetic architecture of complex traits suggests that increasing the sample size and/or the number of phenotypes analyzed does not always produce the anticipated gains in terms of novel loci discovery [2].

In this context, linear mixed models have received considerable attention; their flexibility enables among others estimating trait heritability while adjusting for environmental factors, or accounting for population stratification and cryptic relatedness in mixed-linear-model association studies. This trend is best illustrated by the development of a number of software applications catering for linear mixed model analysis, such as GCTA [3], BOLT-LMM [4], and DISSECT [5], as well as practical statistical methods, including GRAMMAR [6], EMMAX [7], and GEMMA [8].

Among these, it has been demonstrated that the regional heritability mapping (RHM) method [9, 10], which consists in estimating the genetic effect of “windows” (or regions) composed of multiple adjacent SNPs (in contrast to GWAS analysis that focuses on individual SNPs), possesses in a number of cases higher statistical power for the detection of causal loci compared with conventional single-SNP GWAS [10, 11], albeit at the expense of computational power. Despite this, research on the RHM method has been thus far mostly limited to simulation studies [12], software development [5], and application to agricultural settings [13]. This can be explained in large part by the fact that (1) historically, most major advances regarding the application of general mixed model methods to genetics have taken place in the field of animal breeding for the purpose of estimating random genetic effects [14], and their application to human genetics is therefore relatively new, and (2) the substantial discoveries that have resulted from the application of GWAS to newly established large-scale genomic cohorts in recent years have overshadowed the benefits and slowed down the spread of other methods, including RHM, which are generally more complex and computationally demanding than single-SNP mapping methods.

In practice, this means that in spite of the firm theoretical foundation and the simulation studies supporting the ability of the RHM method to identify QTLs that cannot be detected by single-SNP GWAS, its potential for the discovery of new susceptibility loci in human diseases is yet to be fully exploited. In this study, we applied the RHM method to primary biliary cholangitis (PBC) in 5643 Japanese individuals, and identified three new loci at the genome-wide significance level (*STAT4, ULK4*, and *KCNH5*), two of which (*ULK4* and *KCNH5*) have not been found associated with PBC in any population previously. These findings highlight the importance of polygenic model approaches like the RHM method for the discovery of new susceptibility genes alongside traditional GWAS analyses, given that these genes could not be detected with a conventional single-SNP GWAS approach. We tested these three new loci in an independent data set of 491 individuals for replication, and used liver tissue mRNA microarray data to analyze gene expression levels. We also carried out a univariate GCTA-GREML analysis [15] to estimate the common SNP heritability of PBC in the Japanese population.

## Material and methods

### Population and genotyping

This study is based on a high-quality clinical data set forming a representative sample of the Japanese population. The samples used in the discovery and replication data sets were collected nationwide from individuals in the Japan PBC-GWAS Consortium; the discovery and replication data sets represent independent samples. General information about the individuals included in this study is presented in Table S1. For the discovery analysis, sample genotyping was performed by using Japonica v1 (660k SNPs) (Toshiba, Japan) [16, 17] and Axiom Genome-Wide ASI 1 (600k SNPs) (Affymetrix, USA) genotyping arrays, as described in previous papers [18, 19]. All samples used for replication were genotyped with Japonica v1 arrays. Imputation was performed with IMPUTE4 [20] in each SNP array to impute SNPs with no genotype data, by using a phased reference panel of 2049 Japanese individuals from a prospective general population cohort study performed by the Tohoku Medical Megabank Organization, Japan [21]. In the discovery cohort, after imputation we extracted the SNPs with an info score over 0.5 in each array, and the two data sets were merged by using the SNPs common to both genotyping platforms (13.8M SNPs). All chromosome and base-pair positions in this paper are given with regard to the GRCh37 (hg19) assembly.

In both the discovery and replication data sets, quality control procedures were performed per individual and per SNP, by using the following criteria in PLINK v1.90 [22]: individuals call rate ≥ 98%, SNPs call rate ≥ 98%, minor allele frequency ≥ 1%, and consistency with Hardy–Weinberg equilibrium (*P* ≥ 10^−6^). Linkage disequilibrium pruning was also performed by using the --indep-pairwise 50 5 0.8 command. Identity by descent was calculated for each sample pair to remove related samples (pi-hat > 0.1). After these steps, there were 5643 individuals (1953 PBC cases, 3690 healthy controls) and 1,022,240 autosomal SNPs remaining for analysis in the discovery data set. SNP cluster plots were used locally as a visual tool to confirm the genotyping quality of specific SNPs and remove those displaying genotyping issues. A small number of regions that displayed convergence problems or extreme values were also removed from the analysis. In the replication data set, 491 individuals (220 PBC cases, 271 healthy controls) remained for analysis.

### Statistical analysis

Linear mixed model methods were used for analysis. The following model was used to estimate the genome-wide SNP heritability:

$$y = X\beta + u + e$$

with Var(*u*) = $G\sigma _u^2$ and Var(*e*) = $I\sigma _e^2$, where *y* is a vector that represents the PBC phenotypes, *β* is a vector of fixed effects that includes the overall mean, sex, genotyping array, as well as the first ten eigenvectors from principal component analysis (PCA), *u* is a vector of random effects representing the whole (genome-wide) genomic additive effect, and *e* is a vector of residual effects. *X* is the design matrix for the fixed effects, *G* is the whole genomic relationship matrix (GRM) computed by using all SNPs, and *I* is a unit matrix; $\sigma _u^2$ and $\sigma _e^2$ represent the genetic and residual variances, respectively.

The RHM method, which consists in scanning the genome by using windows (regions) of a given number of adjacent SNPs [9], was used to estimate the genetic effect of each region of the genome, as well as their significance compared with the null model (i.e., the model above used to calculate the genome-wide SNP heritability, with no regional component). The model used was as follows:

$$y = X\beta + u + w + e$$

with Var(*u*) = $G\sigma _u^2$, Var(*w*) = $Q\sigma _w^2$, and Var(*e*) = $I\sigma _e^2$, where *w* is a vector of random effects representing the regional genomic additive effect, *Q* is the regional GRM, and $\sigma _w^2$ is the regional genomic variance. A window size of 50 SNPs was used in this study. The windows were shifted by 25 SNPs to create a 50% overlap between adjacent windows.

The GRMs were computed by using all of the SNPs to calculate the whole GRM, and by using the SNPs corresponding to each region of the genome to construct the regional GRMs. The genetic relationship between two individuals *j* and *k* was calculated as follows [3]:

$$g_{jk} = \frac{1}{N}\mathop {\sum }\limits_{i = 1}^N \frac{{(x_{ij} - 2p_i)(x_{ik} - 2p_i)}}{{2p_i(1 - p_i)}},$$

where *x*_*ij*_ and *x*_*ik*_ are the genotypes of the *j*th and *k*th individuals, respectively, at the *i*th SNP; *p*_*i*_ is the frequency of the reference allele at the *i*th SNP; and *N* is the total number of SNPs.

Additionally, a single-SNP GWAS analysis was performed to provide a point of comparison to the results obtained with the RHM method, using a logistic regression model with covariate adjustment for sex, genotyping array, and the first ten principal components. The replication data set was adjusted for sex and the first ten principal components.

### Computer software

The Genetic Complex Trait Analysis (GCTA v1.91.4) software application [3] was used to perform most of the computations and statistical analyses in this study: the computation of the GRMs, the PCA, the univariate Genomic Restricted Maximum Likelihood (GREML) analysis, as well as the RHM analysis. The Average Information Restricted Maximum Likelihood procedure [23] was used for estimation of the variance components. PLINK v1.90 [22] was used to perform quality control as well as the single-SNP GWAS analysis, and R version 3.5.0 [24] was used in combination with the qqman [25] and ggplot2 [26] packages for visualizing the RHM and GWAS results. The limma package v3.42.2 [27] (http://www.bioconductor.org/) was used in R to analyze the mRNA microarray data.

### Significance thresholds

The likelihood ratio test (LRT) statistic, LRT = −2ln(*L*_0_/*L*_1_), was used to test for the presence of regional variance, where *L*_0_ represents the likelihood for the null model (i.e., *H*_0_, with the whole genomic effect but with no regional genomic component), and *L*_1_ represents the likelihood for the alternative model (i.e., *H*_1_, with both the whole and the regional genomic effects). It was assumed that the distribution of the LRT for regional variance follows a 50–50 mixture of chi-square distributions with degrees of freedom equal to 0 and 1 [28].

Bonferroni correction was performed to adjust for multiple testing; to estimate the Bonferroni-corrected significance thresholds, we used half the number of regions tested to account for overlapping windows [9]. The genome-wide critical *P* values and corresponding LRT thresholds are provided in Table 1.

**Table 1:** *P* value and LRT thresholds for the RHM analysis.

	*α* = 0.05	*α* = 0.1
Number of regions	40,890	40,890
Bonferroni-corrected *P* value threshold	2.45E−06	4.89E−06
Corresponding LRT threshold	20.88	19.55

*α* significance level.

### PBC prevalence in Japan

To adjust the heritability estimates for ascertainment the heritability estimates were transformed from the observed scale to the liability scale by using disease prevalence estimates for the general population [1]. Because considerable variation has been reported in the prevalence of PBC between countries, we used prevalence estimates based on nationwide epidemiological studies of PBC in Japan. Concretely, given an estimated range of 380–460 cases per million in the literature [29], we assumed a prevalence of 420 per million. All of the heritability estimates reported in this study are given on the liability scale.

### mRNA data analysis

Liver biopsy was performed to obtain liver tissue samples for the mRNA microarray analysis. All patients for whom liver biopsy specimens were available were included in this analysis; specifically, patients with PBC (36 individuals), chronic hepatitis C (CHC) (15 individuals), and metastatic liver cancer (5 individuals; normal liver tissue). Details about RNA extraction and preservation have been described previously [21]. Quantitative DNA microarray data were obtained using Agilent Feature Extraction software (Agilent Technologies), and data normalization (excluding lincRNA) was performed with the quantile method. Statistical analysis was performed with the limma package [27] in R, the *P* value indicated corresponding to PBC versus CHC/normal samples.

## Results

### Genome-wide SNP heritability

In this study, we calculated the GREML-based common SNP heritability estimate of PBC (±standard error), which is generally thought of as the lower-bound estimate of narrow-sense heritability. It was estimated to be 0.210 (±0.026), adjusting for sex, genotyping array, and the first ten principal components.

### Regional heritability mapping analysis

**Fig. 1:**
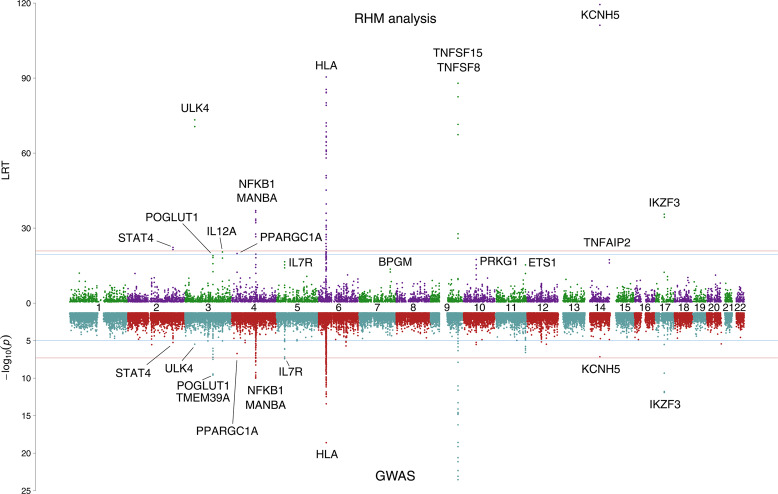
Miami plot of the RHM and GWAS analyses. In the LRT plot of the RHM analysis (top), each dot represents a region of 50 adjacent SNPs; the red and blue horizontal lines represent the significant (LRT > 20.88) and suggestive (LRT > 19.55) thresholds, respectively. In the Manhattan plot of the GWAS analysis (bottom), the red and blue horizontal lines represent the significant (*P* < 5 × 10^−8^) and suggestive (*P* < 10^−5^) thresholds, respectively.

**Fig. 2:**
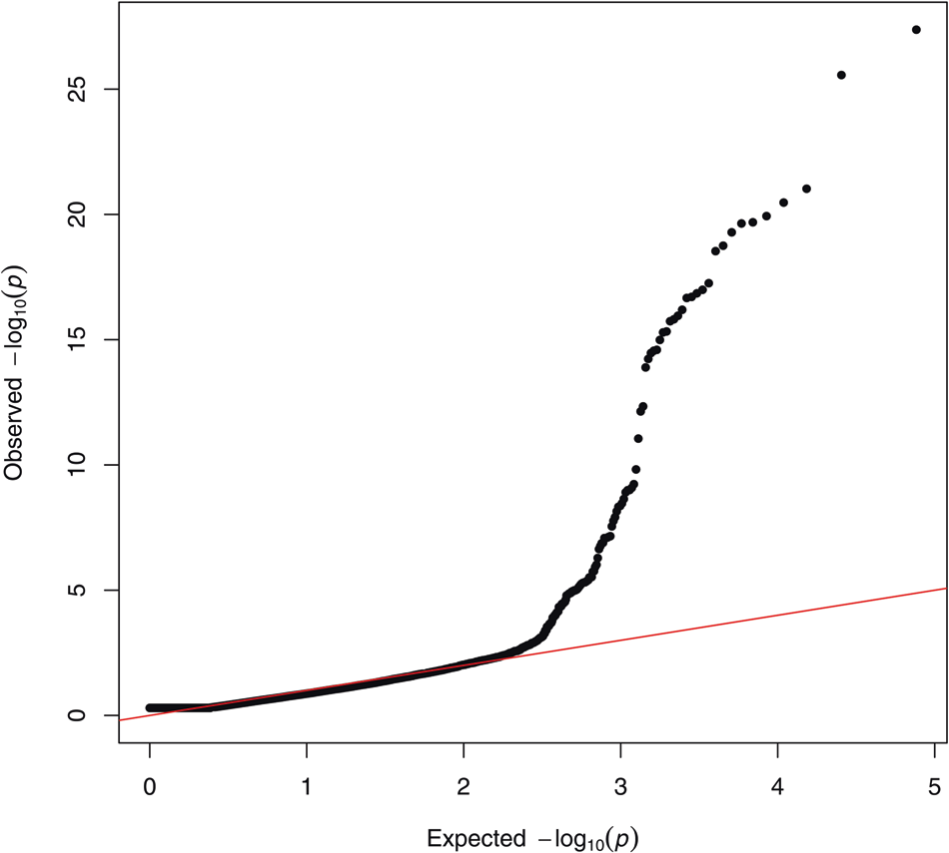
QQ-plot of the RHM analysis. The genomic inflation factor was equal to 1.

The results of the RHM analysis are presented in Fig. 1 (top), which shows the result of the LRT comparing the likelihood of the model including regional genomic effects against the null model (i.e., without regional genomic effects) for each 50-SNP region of the genome. The corresponding QQ-plot is shown in Fig. 2.

The regions that were found to be significant at the genome-wide level (LRT > 20.88) in this analysis are located on the following genes: *STAT4, ULK4, NFKB1/MANBA, HLA, TNFSF15/TNFSF8, KCNH5*, and *IKZF3*. Information about each significant non-HLA region, as well as detail about the corresponding LRT and regional heritabilities, is provided in Table 2. Along with the HLA complex, the *NFKB1/MANBA, TNFSF15/TNFSF8*, and *IKZF3* genes have already been examined in a number of publications on PBC in the Japanese population [18, 30–32] and will therefore not be discussed further here.

**Table 2:** Significant (LRT > 20.88) and suggestive (19.55 < LRT < 20.88) non-HLA regions identified by using the RHM method.

Window	Chr	Position first SNP	Position last SNP	Gene	Min. LRT	Max. LRT	Min. Reg. h^2^	Max. Reg. h^2^	Average SE	Reference
5978–5979	2	191,874,317	192,069,055	STAT4	21.44	22.29	0.00267	0.00271	0.00128	Current study
7437–7438	3	41,587,181	41,710,175	ULK4	70.62	73.30	0.05895	0.06232	0.01316	Current study
11495–11501	4	103,394,414	104,099,060	NFKB1/MANBA	26.56	37.02	0.00239	0.00346	0.00132	Hitomi et al. [19]
25151–25156	9	117,419,622	117,779,933	TNFSF15/TNFSF8	25.97	87.95	0.00285	0.00662	0.00163	Hitomi et al. [19]
33864–33865	14	63,299,030	63,442,462	KCNH5	111.16	119.42	0.10254	0.14534	0.01658	Current study
37167–37168	17	37,497,862	38,698,666	IKZF3	34.37	35.56	0.00296	0.00394	0.00139	Hitomi et al. [19]
9339–9340	3	159,549,003	159,720,504	IQCJ-SCHIP1/IL12A	18.01	20.45	0.00232	0.00238	0.00130	Liu et al. [43]
10282	4	24,176,903	24,243,680	PPARGC1A	19.84	19.84	0.06998	0.06998	0.01662	Inamine et al. [34]

The suggestive regions are given in the lower part of the table. The chromosome and base-pair positions are given with regard to the GRCh37 (hg19) assembly.

*h*^2^ heritability, *LRT* likelihood ratio test for regional heritability > 0, *SE* standard error.

To provide a point of comparison to the results obtained with the RHM method, we performed a single-SNP GWAS analysis of the same data (Fig. 1 (bottom); the corresponding QQ-plot is shown in Fig. S1). Although most genes identified by RHM were also detected by GWAS, the overlap was incomplete as three genes (*STAT4, ULK4*, and *KCNH5*) did not reach genome-wide significance (*P* < 5 × 10^−8^) in the GWAS analysis, and conversely two genes (*POGLUT1* and *IL7R*) were identified by GWAS but not RHM.

The RHM analysis provides evidence, for the first time, of a statistically significant association between *STAT4* and PBC in the Japanese population at the genome-wide level. Beside the discovery of this association, this finding is consequential given that it demonstrates the potential of the RHM method for the detection of new disease-related genes in human populations, as this association would have gone undetected had we only performed a conventional GWAS analysis.

RHM also identified two entirely new susceptibility loci, located on the *ULK4* and *KCNH5* genes. We performed additional analyses for validation and to confirm their involvement in disease pathogenesis by uncovering differences in gene expression through mRNA microarray data analysis (see below).

Additionally, the RHM analysis detected two other loci, *IQCJ-SCHIP1/IL12A* and *PPARGC1A*, at the suggestive level of significance (19.55 < LRT < 20.88). The *IL12A* gene is known to share a common pathogenic pathway with *STAT4* and play an essential role in the development of PBC [33]. Although less is known about *PPARGC1A*, at least two Japanese studies have provided evidence of its function in PBC progression through the regulation of bile acid synthesis [34, 35].

### Replication and differences in gene expression

We used an independent replication data set of 491 Japanese individuals (220 cases, 271 controls) to validate the *STAT4, ULK4*, and *KCNH5* genes. The results, presented in Table 3, confirmed the association of *STAT4* and *KCNH5* with PBC; while the replication of *STAT4* was expected given that its role is known in PBC, the strongest replication was at *KCNH5* (*P* = 5.83 × 10^−25^), a new candidate gene that only RHM detected. This *KCNH5* locus corresponds to rs28608483 (chr14:g.63375059A > G), which had a *P* value of *P* = 7.33 × 10^−8^ (OR = 1.57) in the discovery data set. (The minor allele frequencies in controls and cases were 0.073 and 0.095, respectively).

**Table 3:** Summary statistics of the replication data set.

Chr	SNP	Gene	EA	OR	SE	CI (95%)	*P* value	Adj. *P* value
2	rs7574865	*STAT4*	T	1.436	0.1445	1.082–1.907	0.01217	0.03651
3	rs35391137	*ULK4*	G	1.109	0.3847	0.5216–2.356	0.7886	1
14	rs28608483	*KCNH5*	G	11.16	0.2338	7.057–17.64	5.83E−25	1.749E−24

*CI* confidence interval, *EA* effect allele, *OR* odds ratio, *SE* standard error.

This analysis, however, failed to replicate the findings for *ULK4*. Nevertheless, subsequent mRNA analysis performed to examine differences in gene expression in liver tissues provided promising results. While no gene expression was detected for *KCNH5*, for *ULK4* gene expression levels were significantly higher in PBC patients (*P* < 0.01) (Table S2). This finding is supported by the fact that rs35391137 (chr3:g.41617623A > G), the SNP with the lowest *P* value that we have identified in the *ULK4* region in the discovery data set (*P* = 3.37 × 10^−6^; OR = 1.66), corresponds to the binding site for the Hand1:E47 and Smad3 transcription factors [36].

However, in-depth analyses of the biological pathways of *ULK4* and *KCNH5* are necessary to uncover the mechanisms underlying disease etiology and progression, as well as clarify the potential role of the eQTL effects that have been found for *ULK4*, through cell-specific eQTL analyses of immune cells, in B, CD4+ T cells, CD8+ T cells, monocytes, and natural killer cells in the Japanese population (Table S3) [37].

## Discussion

### The genetic component of PBC

While autoimmune diseases are considered complex and multifactorial, most have been shown to display high heritabilities [38]. PBC is no exception according to the research on familial occurrence and monozygotic twins, which has confirmed that family history is a strong risk factor for developing the disease [39]. Nevertheless, few estimates of the SNP heritability of PBC are available, as most of the publications providing measures of the heritability of PBC are based on family studies, and in such cases it is generally reported in the form of sibling relative risk estimates or differences in concordance rates between monozygotic twins and dizygotic twins [40].

Our estimate of the common SNP heritability of PBC (0.210 ± 0.026) is somewhat low given that previous studies suggest a strong genetic component to PBC, e.g., it is known to have much higher concordance rates in monozygotic twins (63%, one of the highest among autoimmune diseases) than dizygotic twins (≈general population), and a sibling relative risk of 10.5 [39]. However, these results are not contradictory since estimates from twin studies differ in their assessment of environmental components and include effects that common SNP heritability estimates are not meant to capture [41]. For this reason, a direct comparison of estimates is difficult, and more studies are needed in all human populations to further our understanding of the SNP heritability of PBC.

### Identifying *STAT4* in the Japanese population

Although *STAT4* is a well-known PBC risk locus in populations of European ancestry and its role in the pathogenesis of PBC as well as other autoimmune diseases has already been thoroughly examined [42], previous single-SNP GWAS analyses in the Japanese population had failed to replicate this result and hinted at possible genetic differences between populations [18, 31]. In our study, single-SNP GWAS also failed to identify *STAT4* at the genome-wide significance level, while RHM detected an association signal; it is therefore possible that the failure of Japanese GWAS to identify *STAT4* is simply due to the smaller sample sizes of these analyses, or maybe to untagged causal variants.

Although rs7574865 (chr2:g.191964633T > G), the top *STAT4* SNP (*P* = 2.75 × 10^−5^) in a recent study of 2886 Japanese individuals (1381 PBC cases, 1505 healthy controls) [18], reached genome-wide significance in a GWAS performed in 11,375 individuals of European ancestry (2861 cases, 8514 controls) [43], earlier GWAS studies that reported associations between *STAT4* and PBC in populations of European ancestry and that were of a more limited scale, such as those performed by Hirschfield et al. [44] and Liu et al. [45], only provided suggestive evidence of a statistical association between *STAT4* and PBC. While sample size is not the only factor at play—differences exist for instance in terms of minor allele frequency between European and Asian populations with respect to rs7574865, one can assume that future GWAS analyses of PBC with larger sample sizes will detect an association signal at the genome-wide significance level for *STAT4* in the Japanese population. RHM is therefore in all likelihood an effective approach when sample size matters for the detection of a given locus.

### Single-SNP GWAS and RHM: a different set of QTLs?

The fact that *STAT4* was detected by the RHM method but did not reach genome-wide significance in the single-SNP GWAS analysis also corroborates the results from simulations studies demonstrating that RHM often has higher power than single-SNP mapping methods [10, 11], and is therefore a valuable tool for the discovery of new susceptibility loci in human diseases. On the other hand, our GWAS analysis identified two loci that are already known PBC QTLs in the Japanese population, namely *IL7R* (rs7717955 (chr5:g.35862841C > T); *P* = 3.91 × 10^−8^) and *CD80/POGLUT1* (rs13092998 (chr3:g.119245044G > T); *P* = 2.57 × 10^−^^10^) [19, 46], but that were not detected by using the RHM method. Even though computer simulations have previously indicated that GWAS and RHM tend to uncover slightly different sets of QTLs depending on the characteristics of the locus considered (such as the minor allele frequency or the number of QTLs of the region) [11, 12], our findings represent strong empirical evidence that this is indeed the case for human diseases.

Regarding the characteristics of the loci detected by either one of the two methods, our findings seem to be in line with computer simulations showing that RHM has more power than GWAS when it comes to detecting regions with multiple QTLs, but suggest that single-SNP GWAS may be more efficient in some instances when the susceptibility locus contains only a single causal variant [11]. The case of *STAT4*, for example, supports this idea, given that it reached genome-wide significance in our RHM analysis but not with GWAS, and results from previous GWAS analyses have revealed that *STAT4* comprises several independent association signals with PBC [47]. This may explain why single-SNP mapping methods appear to be slightly underpowered for the detection of this type of locus. Conversely, although many SNPs in the *IL7R* gene, which was detected with GWAS but not RHM, have been found to be associated with PBC and other autoimmune diseases, it appears that they are all linked to a single locus, rs6897932 (chr5:g.35874575C > T), known to affect the inclusion of exon 6 through splicing regulation. According to the 1000 Genomes Phase 3 database, the top *IL7R* SNP in our analysis, rs7717955, is in perfect linkage disequilibrium with rs6897932 in the Japanese population (*D*′ = 1; *r*^2^ = 1) [47]. Our findings therefore support the hypothesis that single-SNP GWAS is likely to be more effective than RHM for the detection of loci for which a single QTL is responsible for the regional genetic effect.

Although more discovery studies using the RHM method are needed to confirm the results of our analysis, it seems that while RHM is an effective and practical method for the detection of susceptibility loci that are not easily identified by GWAS, it should be considered as a complementary approach to single-SNP GWAS analysis rather than a potential replacement.

### Replication of RHM results

From a methodological point of view, the process of discovery and replication in GWAS is fairly straightforward: (1) identify a statistically significant SNP in the discovery data set, and then (2) use the same SNP for replication in an independent cohort. The only major caveat is that the SNP to be replicated—or a near-perfect proxy—must exist in the replication cohort. With RHM, several questions arise. First, applying the same standard as single-SNP GWAS—i.e., using the same SNPs in the discovery and replication cohorts—is not realistic in most cases since in RHM a given window contains tens or hundreds of SNPs; replication would therefore entail that all of the SNPs contained in a given window in the discovery cohort must also exist in the replication cohort. A more practical alternative would be to choose windows that cover exactly the same genomic region, i.e. at least the first and last SNPs are the same, with the additional assumption that the SNPs in between capture the regional genomic variance to the same degree in both cohorts. Proceeding this way, however, raises several other issues, such as the difference in marker density between windows in the target cohorts. Another key issue to contend with is sample size, given that replication cohorts are usually much smaller than discovery cohorts. For unrelated individuals and common SNPs, univariate mixed model analyses using REML methodology typically require sample sizes of over 3000 to bring the standard error of the SNP heritability below 0.1 [48], and in many cases, including this study, such thresholds are prohibitive. To validate our results, we therefore chose to use a different approach; among the SNPs contained in the windows identified with RHM in the discovery data set, we selected those with the lowest *P* value in the GWAS analysis—e.g., for *KCNH5* this corresponds to rs28608483 (*P* = 7.33 × 10^−8^ in the discovery data set)—and used them for replication. While we acknowledge that applying different approaches in the discovery and replication data sets is an imperfect strategy, we deemed it the most appropriate in this case.

### Pursuing complementary alternatives to single-SNP mapping methods

On a different note, the findings of our study, by validating simulation results showing that the RHM method is able to identify QTLs that cannot be easily detected by single-SNP GWAS, and by demonstrating that RHM is a powerful tool for the discovery of new susceptibility loci, imply that analytical approaches that focus on the joint effect of multiple SNPs are effective in capturing genetic variation for highly polygenic traits and deserve a more prominent place alongside traditional GWAS methods for the detection of new loci. Our results also suggest that their systematic implementation is desirable whenever possible, especially when statistical power is an issue, for instance when the cost of gathering large amounts of data becomes a major hurdle and/or sample size is limited to begin with, such as in the case of rare diseases or for studies targeting people from a given genetic group for which large data sets are unavailable. More broadly, these findings can be extended to the application of a number of methods other than single-SNP GWAS; even though in this study we chose to focus on RHM for the analysis of common SNPs given the dearth of studies on the application of RHM to human traits for the discovery of new susceptibility loci, other approaches such as SKAT-O for the detection of rare variants are also necessary.

In the specific case of PBC in Japan, the value of pursuing complementary analytic methods in parallel with conventional single-SNP GWAS analyses with larger sample sizes is gaining recognition [49], one of the underlying reasons being that GWAS analyses performed in the Japanese population so far have only managed to identify a small number of susceptibility loci in comparison with European populations. We believe that methods that make use of multiple adjacent SNPs jointly can be particularly useful in this context given the increasing diversity of genetic cohorts worldwide.

## Conclusion

In summary, this study provides strong empirical evidence that RHM is an effective tool for the identification of new susceptibility loci in human diseases with the ability to identify QTLs that cannot be detected with conventional single-SNP mapping methods. We identified associations at the genome-wide significance level between three new loci (*STAT4, ULK4*, and *KCNH5*) and PBC in the Japanese population, two of which (*ULK4* and *KCNH5*) have not been identified in any population previously; this result was obtained by applying RHM and could not be achieved with single-SNP GWAS only. At the same time, this approach is not a replacement for GWAS, as GWAS appears to have its own benefits regarding QTL discovery. Additional research is nonetheless required to deepen our understanding of the underlying mechanisms between these new genes and the development of PBC.

## Web resources

PLINK, https://www.cog-genomics.org/plink/1.9/. GCTA, https://cnsgenomics.com/software/gcta/. R, https://www.r-project.org/. limma, http://www.bioconductor.org/packages/release/bioc/html/limma.html.

## Supplementary information

Supplemental legends

Supplementary Figure 1

Supplementary Table 1

Supplementary Table 2

Supplementary Table 3

## Data Availability

The analyses presented in this study were in part based on data accessed through the Tohoku Medical Megabank Organization (https://www.megabank.tohoku.ac.jp/english/). The summary statistics of the RHM and GWAS reported in this paper are available at the National Bioscience Database Center Human Database (NBDC Human Database; https://humandbs.biosciencedbc.jp/en/) public repository (Research ID: hum0261.v1).

## References

[1] Lee SH, Wray NR, Goddard ME, Visscher PM (2011). Estimating missing heritability for disease from genome-wide association studies. Am J Hum Genet.

[2] Allison SJ (2018). GWAS highlights challenges associated with identification of DKD risk variants. Nat Rev Nephrol.

[3] Yang J, Lee SH, Goddard ME, Visscher PM (2011). GCTA: a tool for genome-wide complex trait analysis. Am J Hum Genet.

[4] Loh PR, Tucker G, Bulik-Sullivan BK, Vilhjalmsson BJ, Finucane HK, Salem RM (2015). Efficient Bayesian mixed-model analysis increases association power in large cohorts. Nat Genet.

[5] Canela-Xandri O, Law A, Gray A, Woolliams JA, Tenesa A (2015). A new tool called DISSECT for analysing large genomic data sets using a Big Data approach. Nat Commun.

[6] Aulchenko YS, Ripke S, Isaacs A, Van Duijn CM (2007). GenABEL: an R library for genome-wide association analysis. Bioinformatics..

[7] Kang HM, Sul JH, Service SK, Zaitlen NA, Kong SY, Freimer NB (2010). Variance component model to account for sample structure in genome-wide association studies. Nat Genet.

[8] Zhou X, Stephens M (2012). Genome-wide efficient mixed-model analysis for association studies. Nat Genet.

[9] Nagamine Y, Pong-Wong R, Navarro P, Vitart V, Hayward C, Rudan I (2012). Localising loci underlying complex trait variation using regional genomic relationship mapping. PLoS ONE.

[10] Shirali M, Knott SA, Pong-Wong R, Navarro P, Haley CS (2018). Haplotype heritability mapping method uncovers missing heritability of complex traits. Sci Rep.

[11] Uemoto Y, Pong-Wong R, Navarro P, Vitart V, Hayward C, Wilson JF (2013). The power of regional heritability analysis for rare and common variant detection: simulations and application to eye biometrical traits. Front Genet.

[12] Caballero A, Tenesa A, Keightley PD (2015). The nature of genetic variation for complex traits revealed by GWAS and regional heritability mapping analyses. Genet.

[13] Gervais O, Pong-Wong R, Navarro P, Haley CS, Nagamine Y (2017). Antagonistic genetic correlations for milking traits within the genome of dairy cattle. PLoS ONE.

[14] Henderson CR (1984). Applications of linear models in animal breeding.

[15] Yang J, Benyamin B, McEvoy BP, Gordon S, Henders AK, Nyholt DR (2010). Common SNPs explain a large proportion of the heritability for human height. Nat Genet.

[16] Kawai Y, Mimori T, Kojima K, Nariai N, Danjoh I, Saito R (2015). Japonica array: improved genotype imputation by designing a population-specific SNP array with 1070 Japanese individuals. J Hum Genet.

[17] Nagasaki M, Yasuda J, Katsuoka F, Nariai N, Kojima K, Kawai Y (2015). Rare variant discovery by deep whole-genome sequencing of 1,070 Japanese individuals. Nat Commun..

[18] Kawashima M, Hitomi Y, Aiba Y, Nishida N, Kojima K, Kawai Y (2017). Genome-wide association studies identify PRKCB as a novel genetic susceptibility locus for primary biliary cholangitis in the Japanese population. Hum Mol Genet.

[19] Hitomi Y, Ueno K, Kawai Y, Nishida N, Kojima K, Kawashima M (2019). POGLUT1, the putative effector gene driven by rs2293370 in primary biliary cholangitis susceptibility locus chromosome 3q13. 33. Sci Rep.

[20] Bycroft C, Freeman C, Petkova D, Band G, Elliott LT, Sharp K, et al. Genome-wide genetic data on ∼500,000 UK Biobank participants. bioRxiv. 2017. 10.1101/166298.

[21] Ueno K, Aiba Y, Hitomi Y, Shimoda S, Nakamura H, Gervais O, et al. Integrated GWAS and mRNA microarray analysis identified IFNG and CD40L as the central upstream regulators in primary biliary cholangitis. Hepatol Commun. 2020. 10.1002/hep4.1497. 10.1002/hep4.1497 PMC7193132 32363322

[22] Purcell S, Neale B, Todd-Brown K, Thomas L, Ferreira MA, Bender D (2007). PLINK: a tool set for whole-genome association and population-based linkage analyses. Am J Hum Genet.

[23] Gilmour AR, Thompson R, Cullis BR (1995). Average information REML: an efficient algorithm for variance parameter estimation in linear mixed models. Biometrics..

[24] R Core Team. R: a language and environment for statistical computing. Vienna: R Foundation for Statistical Computing; 2019. https://www.R-project.org/.

[25] Turner SD. qqman: an R package for visualizing GWAS results using QQ and manhattan plots. bioRxiv. 2014. 10.1101/005165.

[26] Wickham H (2016). ggplot2: elegant graphics for data analysis (Use R!).

[27] Ritchie ME, Phipson B, Wu DI, Hu Y, Law CW, Shi W (2015). limma powers differential expression analyses for RNA-sequencing and microarray studies. Nucleic Acids Res.

[28] Visscher PM (2006). A note on the asymptotic distribution of likelihood ratio tests to test variance components. Twin Res Hum Genet.

[29] Ohira H (2014). Autoimmune liver diseases: perspectives from Japan.

[30] Yasunami M, Nakamura H, Tokunaga K, Kawashima M, Nishida N, Hitomi Y (2017). Principal contribution of HLA-DQ alleles, DQB1* 06: 04 and DQB1* 03: 01, to disease resistance against primary biliary cholangitis in a Japanese population. Sci Rep.

[31] Nakamura M, Nishida N, Kawashima M, Aiba Y, Tanaka A, Yasunami M (2012). Genome-wide association study identifies TNFSF15 and POU2AF1 as susceptibility loci for primary biliary cirrhosis in the Japanese population. Am J Hum Genet.

[32] Hitomi Y, Nakatani K, Kojima K, Nishida N, Kawai Y, Kawashima M (2018). NFKB1 and MANBA confer disease-susceptibility to primary biliary cholangitis via independent putative primary functional variants. Cell Mol Gastroenterol Hepatol.

[33] Joshita S, Umemura T, Nakamura M, Katsuyama Y, Shibata S, Kimura T (2014). STAT4 gene polymorphisms are associated with susceptibility and ANA status in primary biliary cirrhosis. Dis Markers.

[34] Inamine T, Higa S, Noguchi F, Kondo S, Omagari K, Yatsuhashi H (2013). Association of genes involved in bile acid synthesis with the progression of primary biliary cirrhosis in Japanese patients. J Gastroenterol.

[35] Nishida N, Aiba Y, Hitomi Y, Kawashima M, Kojima K, Kawai Y (2018). NELFCD and CTSZ loci are associated with jaundice-stage progression in primary biliary cholangitis in the Japanese population. Sci Rep.

[36] Boyle AP, Hong EL, Hariharan M, Cheng Y, Schaub MA, Kasowski M (2012). Annotation of functional variation in personal genomes using RegulomeDB. Genome Res.

[37] Ishigaki K, Kochi Y, Suzuki A, Tsuchida Y, Tsuchiya H, Sumitomo S (2017). Polygenic burdens on cell-specific pathways underlie the risk of rheumatoid arthritis. Nat Genet.

[38] Selmi C, Lu Q, Humble MC (2012). Heritability versus the role of the environment in autoimmunity. J Autoimmun.

[39] Webb GJ, Siminovitch KA, Hirschfield GM (2015). The immunogenetics of primary biliary cirrhosis: a comprehensive review. J Autoimmun.

[40] Mells GF, Kaser A, Karlsen TH (2013). Novel insights into autoimmune liver diseases provided by genome-wide association studies. J Autoimmun.

[41] Falconer DS, Mackay TFC (1996). Introduction to quantitative genetics.

[42] Lamana A, López-Santalla M, Castillo-González R, Ortiz AM, Martín J, García-Vicuña R (2015). The minor allele of rs7574865 in the STAT4 gene is associated with increased mRNA and protein expression. PLoS ONE.

[43] Liu JZ, Almarri MA, Gaffney DJ, Mells GF, Jostins L, Cordell HJ (2012). Dense fine-mapping study identifies new susceptibility loci for primary biliary cirrhosis. Nat Genet.

[44] Hirschfield GM, Liu X, Xu C, Lu Y, Xie G, Lu Y (2009). Primary biliary cirrhosis associated with HLA, IL12A, and IL12RB2 variants. N Engl J Med.

[45] Liu X, Invernizzi P, Lu Y, Kosoy R, Lu Y, Bianchi I (2010). Genome-wide meta-analyses identify three loci associated with primary biliary cirrhosis. Nat Genet.

[46] Aiba Y, Yamazaki K, Nishida N, Kawashima M, Hitomi Y, Nakamura H (2015). Disease susceptibility genes shared by primary biliary cirrhosis and Crohn’s disease in the Japanese population. J Hum Genet.

[47] Juran BD, Hirschfield GM, Invernizzi P, Atkinson EJ, Li Y, Xie G (2012). Immunochip analyses identify a novel risk locus for primary biliary cirrhosis at 13q14, multiple independent associations at four established risk loci and epistasis between 1p31 and 7q32 risk variants. Hum Mol Genet.

[48] Visscher PM, Hemani G, Vinkhuyzen AA, Chen GB, Lee SH, Wray NR (2014). Statistical power to detect genetic (co)variance of complex traits using SNP data in unrelated samples. PLoS Genet.

[49] Im C, Sapkota Y, Moon W, Kawashima M, Nakamura M, Tokunaga K (2018). Genome-wide haplotype association analysis of primary biliary cholangitis risk in Japanese. Sci Rep.

[50] Ministry of Education, Culture, Sports, Science and Technology, Ministry of Health, Labour and Welfare, Ministry of Economy, Trade and Industry. Ethical guidelines for human genome/gene analysis research (March 2001. Updated February 2013). 2020. Available from: http://www.lifescience.mext.go.jp/files/pdf/n1115_01.pdf (in Japanese).

