# Supplementary material for: Regional heritability mapping identifies several novel loci (STAT4, ULK4, and KCNH5) for primary biliary cholangitis in the Japanese population
Source: Eur J Hum Genet. 2021 Apr 9;29(8):1282–91. doi: 10.1038/s41431-021-00854-5 (PMC8385030; doi:10.1038/s41431-021-00854-5)
Supplement: Supplementary file 1 — Supplemental legends [file 41431_2021_854_MOESM1_ESM.docx]

Supplemental legends

Supplementary Figure 1.

QQ-plot of the single-SNP GWAS analysis. The genomic inflation factor was equal to 1.05.

Supplementary Table 1.

Characteristics of the individuals participating in this study (after quality control).

Supplementary Table 2.

*ULK4* mRNA signal data in liver tissue samples.

Supplementary Table 3.

*ULK4* eQTL variants and their effect sizes used to predict gene expression of B, CD4+ and CD8+ T cells, monocytes, and natural killer cells. (From Ishigaki *et al.,* 2017 [37])
