# Supplementary figures and images for: Regional heritability mapping identifies several novel loci (STAT4, ULK4, and KCNH5) for primary biliary cholangitis in the Japanese population
Source: Eur J Hum Genet. 2021 Apr 9;29(8):1282–91. doi: 10.1038/s41431-021-00854-5 (PMC8385030; doi:10.1038/s41431-021-00854-5)

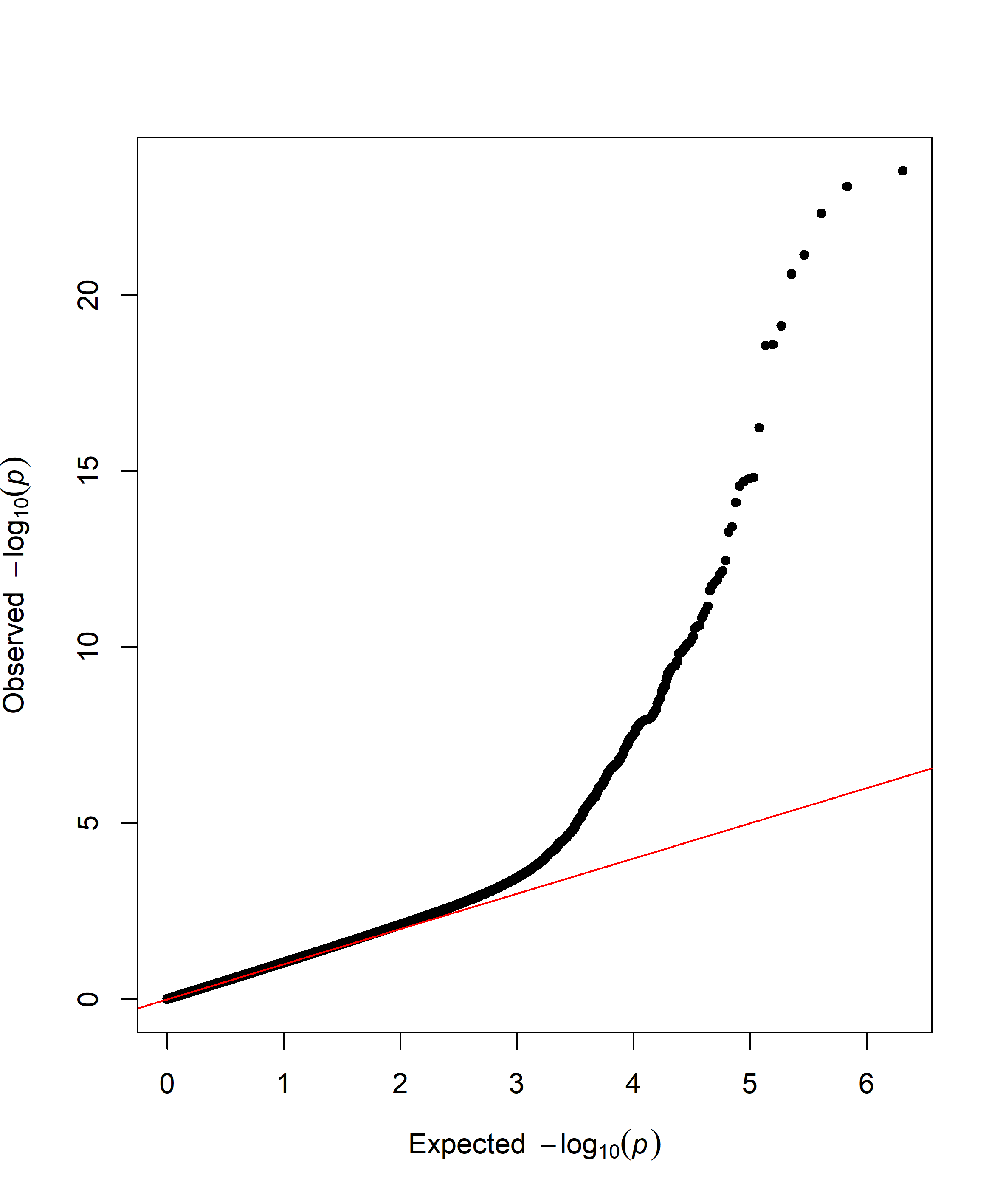

Supplement: Supplementary file 2 — Supplementary Figure 1 [file 41431_2021_854_MOESM2_ESM.tif]
